# Gender-based disparities in the impact of adverse childhood experiences on adult health: findings from a national study in the Kingdom of Saudi Arabia

**DOI:** 10.1186/s12939-017-0588-9

**Published:** 2017-05-30

**Authors:** Maha Almuneef, Nathalie ElChoueiry, Hassan N. Saleheen, Majid Al-Eissa

**Affiliations:** 1 King Abdullah International Medical Research Center and King Saud bin Abdulaziz University for Health Sciences, King Abdulaziz Medical City, Ministry of National Guard - Health Affairs, P. O. Box 22490, Mail code 3202, Riyadh, 11426 Saudi Arabia; 2 National Family Safety Program, King Abdulaziz Medical City, Ministry of National Guard- Health Affairs, Riyadh, Saudi Arabia; 3 Department of Pediatrics, King Abdullah Specialized Children’s Hospital, King Abdulaziz Medical City, Ministry of National Guard -Health Affairs, Riyadh, Saudi Arabia; 4 Department of Pediatrics Emergency, King Abdullah Specialized Children’s Hospital, King Abdulaziz Medical City, Ministry of National Guard -Health Affairs, Riyadh, Saudi Arabia

**Keywords:** Adverse childhood experiences, Kingdom of Saudi Arabia, Mental health problems, Chronic illness, Health risk behaviors

## Abstract

**Background:**

Adverse Childhood Experiences (ACEs) have been linked to an increased risk of health and social problems throughout life. Studies on gender differences from developing countries are scarce. In this paper, we will examine gender variations in the types of reported ACEs and gender-specific relationships between cumulative ACEs and physical and mental health, and Risky Health Behaviors (RHB) in adulthood in the Kingdom of Saudi Arabia (KSA).

**Methods:**

A cross sectional national study was conducted in all of the 13 regions in KSA in 2013 using the ACE- International Questionnaire (ACE-IQ). We used multivariate logistic regression to examine the relationship between 4 + ACEs and physical, mental health and RHBs for both men and women separately after adjusting for age, education, marital status and current employment.

**Results:**

The total number of participants was 10,156 and women comprised 48% of the sample. The majority of respondents (80%) reported at least one ACE. Women had higher percentages of < =2 ACEs (65% vs 55%; *p* <0.05) while men were more likely to have 4+ ACEs (33% vs 25%; *p* < 0.05). When compared to participants with 0 ACE, men who reported 4+ ACEs were associated with the highest likelihood of using drugs (OR = 9.7; 95% CI: 6.4-14.5) and drinking alcohol (OR = 9.2; 95% CI: 6.3-13.6). On the other hand, women who experienced 4+ ACEs were associated with the highest likelihood of depression (OR = 7.0; 95% CI: 5.2-9.4), anxiety (OR = 6.4; 95% CI: 5.0-8.2) and other mental illnesses (OR = 7.4; 95% CI: 5.2-10.6). As for chronic diseases, abused men and women in childhood showed similarly a twofold increased risk of developing diabetes, hypertension, coronary heart disease and obesity when compared to non-abused participants.

**Conclusion:**

Findings highlight the need to consider gender specific differences in the development of preventive strategies to address ACEs in KSA.

## Background

Adverse Childhood Experiences (ACEs) refer to some of the most intensive and frequently occurring sources of stress that children may suffer early in life [1]. Such experiences include multiple types of abuse, neglect, witnessing parental violence, and peer, community, and collective violence. Global estimates show that six in ten people in the general population have been exposed to at least one ACE [2]. Traumatic experiences in childhood have life-long consequences and can disrupt early brain development and increase the risk of a range of physical and mental health disorders [3]. A growing body of epidemiological evidence has consistently shown that ACEs are associated with a wide range of unhealthy behaviors, negative health outcomes, increased health care utilization, and even premature deaths [2, 4–8]. The burden of child maltreatment in its various forms is not limited to its long-term sequel on individual physical and mental health; society also incurs a substantial economic burden. The lifetime cost for each surviving victim of child maltreatment is estimated to be US$210,012, which is comparable to other costly health conditions such as type 2 diabetes, the cost of which is estimated to be between US$181,000 and US$253,000, according to a report published by the Centers for Disease Control and Prevention (CDC) [9].

Differences have been seen in the prevalence of ACEs between men and women. Girls are more likely to experience sexual abuse and to be affected by parental psychiatric problems. However, boys are more likely to report childhood verbal abuse, parental divorce, parental unemployment, and parental death [10, 11]. Gender specific differences have also been reported in the impact of ACEs on negative health outcomes [10–13]. Sexual and verbal abuse during childhood were significantly associated with over smoking for women but not for men [12]. Similarly, hopelessness during adulthood was also significantly associated with ACEs only among women but not among men [13]. In contrast, other researchers have not observed gender specific differences. In a study exploring the effect of ACEs on overall health, there were no statistically significant differences between men and women in depressive symptoms, and tobacco, alcohol, and marijuana use [14].

The majority of ACE studies have been conducted in industrialized countries whereas the prevalence and burden of ACEs and chronic diseases are larger in developing countries [15]. In KSA for example, the Global Burden of Disease 2010 study [16], reported that elevated body mass index was the leading risk factor for Disability Adjusted Life Years (DALY). Dietary risks, high blood pressure, high fasting plasma glucose, physical inactivity, smoking, and drug and alcohol use were listed in the top 10 risk factors for the highest disease burdens in KSA [16]. Being aware of the toll of these diseases and risk factors on the health and economic sectors of the country, the National Family Safety Program (NFSP) implemented in 2012 a pilot ACE study in Riyadh [5] that found 82% of the 931 adult participants experienced one or more ACEs and a third (32%) reported experiencing four or more ACEs. It was also revealed in this pilot study that Saudi women were less likely to experience 4 + ACEs than their men counterparts (29.8% vs 35%). To confirm this pattern, a national ACE study was conducted in 2013 in all regions of the country; this paper is part of that study where we will examine gender variations in the types of reported ACEs and gender specific relationships between cumulative ACEs and physical and mental health and RHBs in adulthood in KSA.

## Methods

### Sample and procedure

In 2013, a cross-sectional study was conducted in all regions/provinces of KSA to identify the prevalence of ACEs and their association with health risk behaviors and chronic diseases among adults aged 18 years or above. Within each region, cities were selected randomly considering both large and small cities (category A and B cities) depending on the size of the region in order to get a representative sample of the entire population. Inclusion criteria for the study were as follows: being a Saudi citizen; residence in a selected city/province; age ≥18 years; and cognitive ability to complete the questionnaire or participate in a face to face interview. The study design was approved by the Institutional Review Board (IRB) of King Abdullah International Medical Research Center (KAIMRC), the governing ethical body for the National Family Safety Program (NFSP).

Surveys were collected from participants in 182 locations all over the kingdom. The questionnaire was handed out to eligible participants, answered in a private place, placed in a sealed envelope after completion, and dropped by participants in a closed box. Before initiating the interview, data collectors informed eligible participants about the goals of the study, explained its voluntary and anonymous nature, and obtained their written consent. It was a self-administered questionnaire; however, a few face-to-face interviews were conducted with those who were unable to read/write. After completing the questionnaire, participants obtained educational materials about the impact of ACEs. The questionnaire took an average of 20 min to complete.

### Measurement of adverse childhood experiences

The study used an established validated survey tool: the Adverse Childhood Experience International Questionnaire (ACE-IQ). This tool was developed by research teams from the World Health Organization (WHO), CDC, and world experts. The questionnaire was translated into Arabic and back translated and the language was modified for cultural adaptability. It was also pilot tested earlier in KSA [5], and the final version of the tool was approved by the national research team. The tool was also checked for internal validity and posted on the WHO website http://www.who.int/violence_injury_prevention/violence/activities/adverse_childhood_experiences/en/. Briefly, there were five different ACE domains examined in the questionnaire that included abuse, family dysfunction, peer, community, and collective violence. Under the five domains there were 13 different ACEs that included (1) emotional abuse, (2) physical abuse, (3) sexual abuse, (4) neglect, (5) household substance use, (6) household mental illness, (7) witnessing domestic violence, (8) incarcerated household member, (9) parental divorce/separation, (10) lack of protection and supervision, (11) peer violence, (12) community violence, and (13) exposure to collective violence before age 18. Each ACE contains different questions with a total of 44 questions. These questions consist of multiple-choice responses: “many times,” “few times,” and “never.” “Many times” and “few times” were combined in order to have a positive response to each ACE. ACE score was generated by summing all the ACEs that participants reported (range 0 to 13) and was categorized into five groups for analysis (0, 1, 2, 3, and ≥4 ACEs).

The ACE-IQ was supplemented with a section (22 items) assessing the presence of physician diagnosed *chronic physical diseases*, *mental health*, and *RHB*s. The most common chronic diseases, namely, diabetes, hypertension, coronary heart disease (CHD) and obesity, were selected. Three dependent mental health variables were used in this study: depression, anxiety, and other mental illness. These questions started with *“Have you ever been diagnosed by a doctor with-------.”* Lifetime engagement in RHBs including smoking (current or previous), regular alcohol consumption, drug use (current or previous), pre or out of wedlock sex, and regular exercise (at least 3 times per week) were also assessed (Table 1). Participants had the option of answering “yes,” “no,” or “refuse” to these questions.

**Table 1:** Questions on physician diagnosed chronic physical diseases, mental health and risky health behaviors

Chronic diseases
Have you ever been diagnosed by a doctor with diabetes?
Have you ever been diagnosed by a doctor with hypertension?
Have you ever been diagnosed by a doctor with coronary heart disease?
Have you ever been diagnosed by a doctor with obesity?
Mental health
Have you ever been diagnosed by a doctor with depression?
Have you ever been diagnosed by a doctor with anxiety?
Have you ever been diagnosed by a doctor with mental illness (other than depression or anxiety)?
Risky health behaviours
Are you a current or previous smoker?
Are you currently or have you previously been drinking alcohol on a regular basis?
Are you currently or have you previously been using recreational drugs?
Do you regularly exercise at least three times a week?
Have you ever had any pre-marital or out of wedlock sexual relations?

Covariates for these analyses included age, sex, marital status (married, previously married, single), current employment (employed, homemaker, student, unemployed/retired), and highest educational attainment (did not graduate from high school, graduated from high school, graduated from college or technical school).

### Ethics

In this study, data was collected only for research purposes and was not used for other purposes. Multiple measures were adopted to ensure confidentiality of data and protect participants. Data collectors received training and continuous monitoring in areas related to ethical conduct, confidentiality protection, and other issues in human subject protection. The survey did not contain the name of the participant but instead was labeled with a reconstructable personal alphanumeric identifier. All data were stored in a password-protected database on computers in locked offices at the National Family Safety Program (NFSP). The hard copy of this information was stored in a locked cabinet that does not contain other data.

### Overview of analysis

Analyses were undertaken using SPSS v20.0 [17]. A significance level of 0.05 was used for all statistical tests. Data were presumed to be missing at random and listwise deletion was employed to handle missing data. Bivariate analyses using chi square tests were undertaken to examine the relationship between gender and prevalence of ACEs. We performed analyses stratified by gender using multivariate logistic regression to examine the relationship between 4 + ACEs (independent variable) and physical and mental health, and RHBs as the dependent variables (1 = Yes and 0 = No). We included in the model the following relevant covariates: age, education, marital status, and current employment, which were expected to impact the dependent variables.

## Results

The sample consisted of 10,156 participants where 52% were men and 48% were women from all 13 regions in KSA. The distributions of selected demographic characteristics of the sample are presented by gender (Table 2). The participants’ mean age was 34.3 ± 11.3 years, 41% had a college degree or higher, 51% were employed, and 58% were married. The majority of women (41%) were housewives, and men were mostly (71%) employed.

**Table 2:** Socio-demographic characteristics of the participants (10,156) in the national ACE study in KSA by gender*

Variables	Men (*n* = 5285) *N* (%)	Women (*n* = 4850) *N* (%)	Total (*n* = 10156) *N* (%)
Socio demographics			
Age (mean)			
Mean ± SD	34.3 ± 11.2	34.2 ± 11.5	34.3 ± 11.3
Education			
< High school	1055 (20)	1175 (24)	2236 (22)
High school	2052 (39)	1599 (33)	3657 (36)
≥College	2106 (40)	2037 (42)	4152 (41)
Employment			
Employed	3745 (71)	1432 (29)	5182 (51)
Homemaker	45 (1)	2006 (41)	2056 (20)
Student	767 (14)	844 (17)	1615 (16)
Unemployed/Retired	610 (12)	445 (9)	1058 (10)
Marital status			
Married	3117 (59)	2775 (57)	5903 (58)
Single	1924 (36)	1477 (30)	3407 (33)
Divorced/Separated/Widowed	200 (4)	535 (11)	739 (7)

*Percentages may not add to 100 due to missing data

### ACE prevalence by gender

The overall majority of respondents (80%) reported at least one ACE before the age of 18 and 29% reported ≥4 ACEs. Distributions of individual ACEs and ACE scores are presented by gender (Table 3). Men were significantly more likely than women to have experienced four or more ACEs (33% vs 25%). Women had significantly higher percentages of < =2 ACEs (65% vs 55%). Men reported higher percentages of childhood psychological abuse (21% vs. 17%, *p* < 0.001), physical abuse (16% vs. 11%, *p* < 0.001), and sexual abuse (16% vs. 11%, p < 0.001). Men also were significantly more likely to experience peer violence (28% vs. 15%, *p* = 0.003), community violence (21% vs. 14%, *p* < 0.001), and collective violence (25% vs. 15%, *p* < 0.001). In terms of household dysfunction adversities, men were significantly more likely to report witnessing domestic violence in childhood (49% vs. 45%, *p* < 0.001), living with a substance abusing household member (12% vs. 7%, *p* < 0.001), and living with an incarcerated household member (12% vs. 10%, *p* = 0.002). In contrast, women reported higher percentages of experiencing parental divorce or separation (25% vs 21%, *p* < 0.001) and living with a household member with mental illness (11% vs 9%, *p* = 0.009).

**Table 3:** Prevalence of individual adverse childhood experiences and ACE scores in KSA by gender*

Adverse childhood experiences	Men	Women	Total
	*N* (%)	*N* (%)	*N* (%)
1. Emotional/psychological abuse	1135 (21)	836 (17)	1974 (19)
2. Physical abuse	859 (16)	513 (11)	1374 (13)
3. Sexual abuse	861 (16)	552 (11)	1414 (14)
4. Neglect (physical/medical/general)†	707 (13)	643 (13)	1357 (13)
5. Household member alcoholic/drug user	597 (12)	347 (7)	945 (10)
6. Household member had depression/mental illness/suicidal	468 (9)	512 (11)	983 (10)
7. Household member was incarcerated	611 (12)	476 (10)	1089 (11)
8. Parents divorced/separated/died	1141 (21)	1236 (25)	2382 (23)
9. Domestic violence in household	2611 (49)	2187 (45)	4808 (47)
10. Lack of protection and supervision	1683 (32)	1679 (35)	3368 (33)
11. Peer violence	1493 (28)	714 (15)	2209 (22)
12. Community violence	1090 (21)	697 (14)	1791 (18)
13. Collective violence	1298 (25)	731 (15)	2031 (20)
ACE score			
0/no exposure	961 (18)	1089 (22)	2054 (20)
1	1154 (21)	1204 (25)	2315 (23)
2	833 (16)	876 (18)	1763 (17)
3	586 (11)	488 (10)	1077 (11)
4+	1751 (33)	1193 (25)	2947 (29)

*Percentages may not add to 100 due to missing data

†*P* values were <0.05 for all except for neglect

### Prevalence of health outcomes and RHBs by gender

Table 4 shows the prevalence of chronic diseases, mental health problems, and RHBs in general by gender. Compared to men, women were significantly more likely to report mental health problems including depression (14% vs 11% *p* < 0.001) and anxiety (21% vs 16%, *p* < 0.001). Additionally, women were significantly more likely to report chronic diseases including diabetes (19% vs 17%, p = 0.003), hypertension (24% vs 18% *p* < 0.001), CHD (7% vs 5%, *p* < 0.001), and obesity (5% vs 3%, *p* < 0.001). However, men were significantly more likely to smoke (57% vs. 17%, *p* < 0.001), drink alcohol (12% vs. 5%, *p* < 0.001), use drugs (11% vs. 5%, *p* < 0.001), and have pre marital or out of wedlock sexual relationship (26% vs. 8%, *p* < 0.001).

**Table 4:** Prevalence of chronic diseases, mental health and risky health behaviors by gender in KSA (*n* = 10,156)*

	Men (*n* = 5285) *N* (%)	Women (*n* = 4850) *N* (%)	Total (*n* = 10156) *N* (%)	*P* value
Chronic diseases				
Diabetes	834 (17)	890 (19)	1727 (18)	0.003
Hypertension	909 (18)	1115 (24)	2027 (21)	*p* < 0.001
Coronary heart disease	265 (5)	325 (7)	592 (6)	0.001
Obesity	173 (3)	245 (5)	420 (4)	*p* < 0.001
Mental health				
Depression	569 (11)	665 (14)	1239 (13)	*p* < 0.001
Anxiety	797 (16)	998 (21)	1801 (19)	*p* < 0.001
Other Mental illness	472 (10)	465 (10)	939 (10)	0.318
Risky health behaviors (lifetime)				
Smoking	2803 (57)	798 (17)	3609 (37)	*p* < 0.001
Regular alcohol consumption	595 (12)	221 (5)	818 (8)	*p* < 0.001
Using illicit drugs	530 (11)	228 (5)	761 (8)	*p* < 0.001
Not doing regular exercise	3656 (74)	3727 (80)	7397 (77)	*p* < 0.001
Pre-marital/out of wedlock sexual relationship	1191 (26)	358 (8)	1552 (17)	*p* < 0.001

*Percentages may not add to 100 due to missing data

### Multivariate results for the effects of ACEs scores on health problems and RHB in adulthood

We examined gender specific relationships of the impact of cumulative ACE on physical and mental health, and RHBs after taking into account the confounding effects of age, education, marital status, and employment. The accumulated number of adverse childhood experiences was significantly associated with each assessed outcome, however, significant gender differences were noted.

**Fig. 1:**
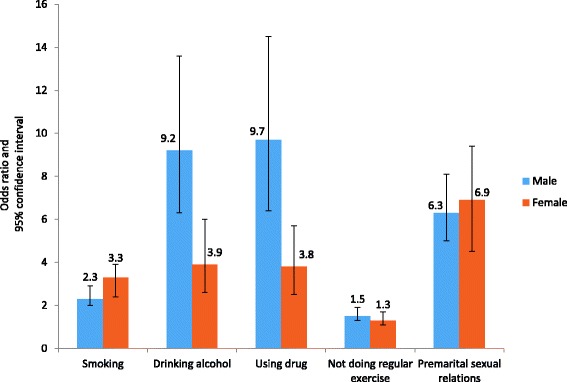
Cumulative effects (4+ ACEs score)* on risky health behaviors: stratified analysis of men and women in KSA †Legend: *Adjusted odds ratios for the socioeconomic factors: age, education, marital status and employment. † All associations are significant at *p* < 0.05

**Fig. 2:**
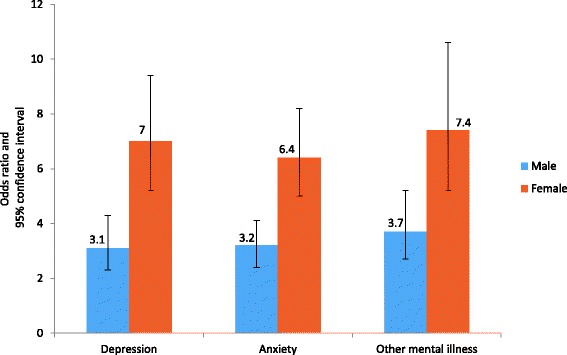
Cumulative effects (4+ ACEs score)* on mental health outcomes: stratified analysis of men and women in KSA† Legend: *Adjusted odds ratios for the socioeconomic factors: age, education, marital status and employment. † All associations are significant at *p* < 0.05

**Fig. 3:**
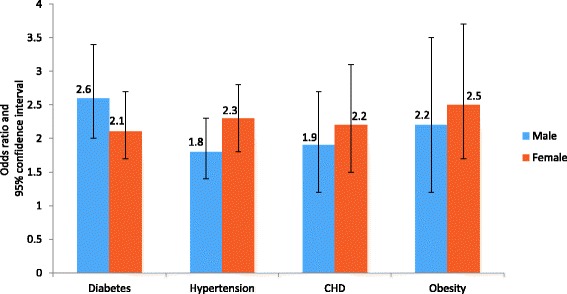
Cumulative effects (4+ ACEs score)* on chronic diseases: stratified analysis of men and women in KSA† Legend: *Adjusted odds ratios for the socioeconomic factors: age, education, marital status and employment. † All associations are significant at *p* < 0.05

Table 5 shows logistic regression for 4 + ACEs and chronic diseases, mental health, and RHBs. When compared with participants with 0 ACE, men who reported 4+ ACEs had the highest likelihood of using drugs (OR = 9.7; 95% CI: 6.4-14.5) and drinking alcohol (OR = 9.2; 95% CI: 6.3-13.6). For women, the odds ratio for using drugs was 3.8 (95% CI 2.5-5.7) and 3.9 (95% CI: 2.6-6.0) for drinking alcohol (Fig. 1). On the other hand, women who experienced 4+ ACEs had the highest likelihood of depression (OR = 7.0; 95% CI: 5.2-9.4), anxiety (OR = 6.4; 95% CI: 5.0-8.2), and other mental illnesses (OR = 7.4; 95% CI: 5.2-10.6). For men, the odds ratio of reporting depression was 3.1 (95% CI 2.3-4.3), 3.2 (95% CI 2.4-4.1) for anxiety, and 3.7 (95% CI 2.7-5.2) for other mental illnesses (Fig. 2). Regarding chronic diseases, abused men and women similarly showed a twofold increased risk of developing physical illnesses in adulthood when compared to non-abused participants. For women who experienced 4+ ACEs, the odds ratio of developing diabetes was 2.1 (95% CI 1.7-2.7), 2.3 (95% CI 1.8-2.8) for hypertension, 2.2 (95% CI 1.5-3.1) for CHD, and 2.5 (95% CI 1.7-3.7) for obesity. For men, the odds ratios for developing diabetes, hypertension, CHD, and obesity were 2.6 (95% CI 2.0-3.4), 1.8 (95% CI 1.4-2.3), 1.9 (95% CI 1.2-2.7), and 2.2 (95% CI 1.3-3.5) respectively (Fig. 3).

**Table 5:** Adjusted Odds Ratios for the impact of ACEs (4+ ACEs score)^†^ on chronic diseases, mental health, and risky health behaviors in KSA: Stratified analysis of men and women

	Men	Women
	OR (95% CI)	OR (95% CI)
Chronic diseases		
Diabetes	2.6 (2.0 - 3.4)	2.1 (1.7 - 2.7)
Hypertension	1.8 (1.4 - 2.3)	2.3 (1.8 - 2.8)
CHD	1.9 (1.2 - 2.7)	2.2 (1.5 - 3.1)
Obesity	2.2 (1.2 - 3.5)	2.5 (1.7 - 3.7)
Mental Health		
Depression	3.1 (2.3 - 4.3)	7.0 (5.2 - 9.4)
Anxiety	3.2 (2.4 - 4.1)	6.4 (5.0 - 8.2)
Other mental illness	3.7 (2.7 - 5.2)	7.4 (5.2 - 10.6)
Risky health behaviors		
Smoking	2.3 (2.0 - 2.9)	3.3 (2.4 - 3.9)
Drinking alcohol	9.2 (6.3 - 13.6)	3.9 (2.6 - 6.0)
Using drug	9.7 (6.4 - 14.5)	3.8 (2.5 - 5.7)
Not doing regular exercise	1.5 (1.3 - 1.9)	1.3 (1.1 - 1.7)
Premarital sexual relations	6.3 (5.0 - 8.1)	6.9 (4.5 - 9.4)

^†^Adjusted for age, education, marital status, and employment

## Discussion

This analysis of representative population-based data in all geographic regions of KSA indicates that childhood abuse was more prevalent among men than women. We also found that experiences of childhood adversity and household dysfunction are related to poor physical, mental, and behavioral health in adulthood. However, the association between ACEs and mental and behavioral health varied for women and men. For women, after taking into accounts all covariates, an increased reporting of ACE was associated with elevated prevalence of psychological and mental disorders. For men, early adversity was associated with a higher risk of substance dependence (alcohol/drug abuse). No noteworthy gender specific differences were noted in regarding to the impact of ACE on all assessed chronic diseases (diabetes, CHD, hypertension and obesity) and remaining RHBs including premarital sexual relationships, smoking and lack of exercise.

Data from the current study indicates that the cumulative ACEs (4+) were more frequent among men (33%) compared to women (25%). Our results are consistent with other regional studies that used the same questionnaire and revealed that men reported higher prevalence of cumulative ACEs (5+) than their women counterparts (11.2% vs 5.1%) [18]. However, other international studies found a higher prevalence of childhood abuse among women compared to men [19–21]. Such differences may be attributable to cultural and regional variations.

Our results are consistent with existing international literature that demonstrates an elevated risk of psychological disorders among women following childhood abuse compared to men [22–25]. Previous studies have revealed that female gender is a significant independent predictor of PTSD [26] and traumatized women are twice as likely to develop PTSD when compared with men [27]. Childhood victimization tends to be associated with higher psychiatric risk among adult women than men [28]. Researchers explain that women are more inclined to blame themselves or be blamed following victimization and are more likely to manifest internalizing behaviors compared with men after being abused [29]. It is also speculated that the tendency to internalize stress symptoms and dwell on negative emotions is more pronounced among women in the region given the high restrictions imposed on them and cultural factors such as norms, values, and ascribed roles. Female victims of abuse in the Arab world particularly may have to suffer their abuse in silence when victimized by a family member, so as not to tarnish their honor or family reputation, or break family unity [30]. In KSA, previous community-based studies and meta-analyses have documented higher rates of mental illnesses among women than men [31, 32] and some researchers have related this to childhood exposure to adverse experiences [33]. A recent study has revealed a relatively high percentage of depression (30%) among adolescent Saudi females and found it to be significantly related to household dysfunction and physical and emotional abuse [33].

Similar to previous international data, our results reveal that abused men in childhood were more likely to display externalizing behaviors and conduct disorder, including drug abuse and alcohol dependence [23, 34, 35]. Schilling et al. (2007) [36] reported that men who experienced ACEs were more likely to engage in antisocial behavior in adulthood than women who experienced similar ACEs. Although drinking alcohol and taking drugs are prohibited in KSA, it is evidently easier for men to find ways to perform such behaviors as maladaptive coping mechanisms to deal with stress resulting from childhood victimization [4]. Such results may provide further evidence of the strong and pernicious impact of childhood adversities, taking into consideration social, religious, and cultural factors in a reserved Middle Eastern country.

Our results suggest that early adversity was significantly associated with all chronic diseases, and experiencing 4+ ACEs increased the risk of having chronic diseases by two-fold for both genders when compared to non-abused adults. However, other unexamined confounding factors (e.g. diet and genetic factors, sports, and disease history) that can contribute to the development of chronic diseases could have affected this relationship [37]. Therefore the impact of these possible confounders on the relationship between ACEs and chronic diseases is unknown and cannot be determined from this study.

A major advantage of this study is that besides collecting detailed data on childhood abuse, it provided national estimates for studied health related conditions and HRBs in KSA by gender. While there have been small scale studies that have examined the prevalence of substance abuse [38, 39] and psychological disorders in the kingdom [31], only a few studies have been implemented at the national population level. Careful consideration should be given to the plausible causes of such acts that appear to be strongly related to adversities during childhood. In KSA, many governmental parties in collaboration with international organizations are making efforts to reduce substance abuse [40, 41] and mental health problems [42] but recent reports have shown trends for increasing rates of drug abuse, smoking, and mental illnesses during previous years [43, 44]. Thus, the findings of this study should provide convincing reasons to start investing in and adopting interventions aimed at decreasing adversities in childhood and promoting healthy parenting behaviors.

### Strength and limitation of the study

To the best of our knowledge, this research is one of the few published reports in the Middle East among adults who reported ACEs and identified the sex-specific relationships between cumulative ACEs and physical and mental health, and RHB in adulthood. This research utilizing a validated instrument was conducted with a nationally representative sample in KSA. The findings of this study can be a baseline information to government to implement child maltreatment prevention program in order to decrease the burden of chronic diseases, mental health, and RHBs. However, this study has a number of limitations that should be used when extrapolating the findings. First, ACE variables and health outcomes were derived from retrospective self-reports that could introduce inaccurate responses and are susceptible to recall bias because of the time that passed since childhood abuse. Thus, participants may not have disclosed abuse that did occur, especially those who experienced abuse when they were too young to remember it. Previous follow up studies that compared retrospective reports with fully documented abuse in childhood have revealed that false negatives rather than false positives are the rule which may lead to underestimates of ACEs [45, 46]. Second, study outcomes are general indicators of health and wellbeing rather than specific measures of physical or mental diseases. Third, as this is a cross-sectional study, it cannot determine causal relationship and reveals only associations. Fourth, our data suggests that ACEs are associated with physical health outcomes but don’t address other potential confounders that might affect this relationship including genetic factors, physical exercise and diet. Furthermore, this study didn’t have data on the duration of exposure to the assessed adversities and the age at which they occurred. Therefore, the association may have been stronger if “duration of exposure” or “age of occurrence of ACE” had been controlled for.

## Conclusion

This study presents interesting gender specific findings and relationship between cumulative ACEs and negative health related outcomes using population-based data in a developing country.

Findings highlight the need to consider gender specific differences in the development of preventive strategies to address ACEs in KSA. Substance abuse prevention programs and policies could be targeted at boys who experienced ACEs; similarly, awareness campaigns aiming at reducing mental health disorders could target girls with a history of early adversity. Furthermore, general evidence based prevention programs such as parenting programs, early sexual education and home visitation programs are needed to be implemented in all the regions of KSA. Finally, future longitudinal studies are needed to confirm the causal relationship between ACEs and health outcomes.

## Abbreviations

ACE-IQ: Adverse childhood experiences international questionnaire
ACEs: Adverse childhood experiences
CDC: Centers for disease control and prevention
CHD: Coronary heart disease
DALY: Disability adjusted life years
KAIMRC: King Abdullah International Medical Research Center
KSA: Kingdom of Saudi Arabia
NFSP: National family safety program
PTSD: Post-traumatic stress disorder
RHB: Risky health behaviors
WHO: World Health Organization
